# Fuzzy logic systems and medical applications

**DOI:** 10.3934/Neuroscience.2019.4.266

**Published:** 2019-10-22

**Authors:** Elena Vlamou, Basil Papadopoulos

**Affiliations:** Department of Civil Engineering, Democritus University of Thrace, Xanthi, 67100, Greece

**Keywords:** fuzzy logic systems, neuro-fuzzy networks, fuzzy logic inference, fuzzy controllers, Parkinson's disease

## Abstract

The combination of Artificial Neural Networks and Fuzzy Logic Systems enables the representation of real-world problems via the creation of intelligent and adaptive systems. By adapting the interconnections between layers, Artificial Neural networks are able to learn. A computing framework based on the concept of fuzzy set and rules as well as fuzzy reasoning is offered by fuzzy logic inference systems. The fusion of the aforementioned adaptive structures is called a “Neuro-Fuzzy” system. In this paper, the main elements of said structures are examined. Researchers have noticed that this fusion could be applied for pattern recognition in medical applications.

## 1. Introduction

This paper highlights the potential uses of fuzzy network structures in the field of medicine and in particular, it focuses on the several methods in which those system in combination with fuzzy logic techniques could be utilized in order to enhance pattern recognition efficiency. Although medicine and control engineering are not directly related, the use of accessible control techniques for on-line devices, particularly in cases of surgical operations and in intensive care units is now feasible. Currently, the application areas of control engineering in medicine range from simple dosage prescription schemes to highly sophisticated adaptive controllers. Real world knowledge can be regarded as incomplete, inaccurate, and inconsistent. Exact medical entities such as fuzzy-sets can be explained by fuzzy logic theory [1]. As it will be reviewed in the following sections, studies have shown that fuzzy logic methodologies can be utilized in early diagnosis of diseases such as Parkinson's disease. Early diagnosis has been proven to be very valuable in creating a more effective treatment plan. Therefore, identifying a method that would allow for early disease diagnosis would be extremely beneficial for the patients. The main contribution of this paper is to analyze various types of fuzzy systems and examine their potential applications in early diagnosis or disease classification.

## 2. Biological and artificial neural networks

The attempts to substitute certain brain cognitive functions by a computer system are not hindered by the existing differences between the structure of the human brain and that of a computer. Artificial Intelligence is employed for the creation and application of systems that imitate not only logical thinking and behavior but also human intelligence [2].

**Figure 1.**
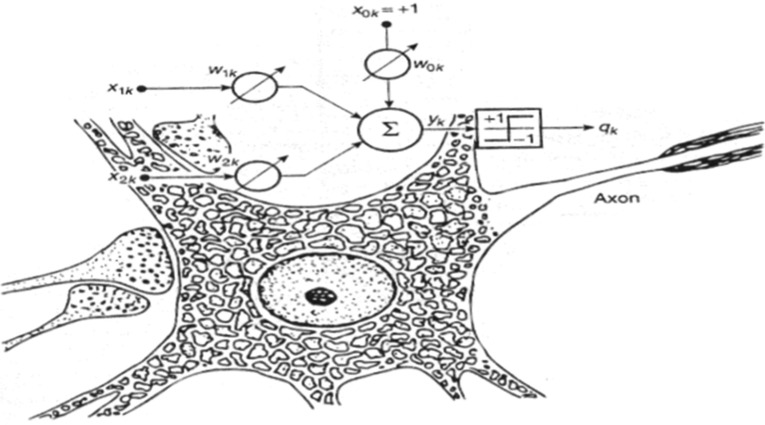
Biological and Artificial Network Simulation.

A number of issues linked with the evolutionary theory arose, when the idea that the human mind could be perceived as a computer whose processes are observed via reverse engineering was formed. The evolutionary theory states that living species evolve over time [3]. However, a series of adaptive variations result in certain evolutionary changes regarding brain functions. Computer simulation that uses computational models consisting of mathematical equations are utilized for the research of cognitive function processes [4]. Such models include but are not limited to artificial neural networks, which as the name suggests were inspired by biological neural networks. In biology, a neuron is the smallest part of the brain and it constitutes the basic difference between animals and plants (plants do not have neurons). A neuron's main function is to process information. In the cortex of the brain there are approximately 10 billion neurons and 60 trillion connections. As it is shown in Figure 1, the main sections of a neuron include the body, the axis and the dendrites, which receive signals from neighboring neurons [5].

## 3. Fuzzy logic

Fuzzy logic–fuzzy systems comprise one of the three pillars of Computational intelligence which in turn is categorised under the broad field of artificial intelligence. The other two pillars are artificial neural networks and evolutionary computing (evolutionary computation). Fuzzy systems, which utilize fuzzy sets and fuzzy logic, are an attempt to effectively describe the uncertainty of the real world. Fuzzy logic is a generalisation of classical logic and provides mechanisms of approximation (approximate reasoning) and inference (decision making). The approximate reasoning is an attempt to model the human way of thinking and inference, as it is known that the human brain performs more approximate considerations based on qualitative criteria of perception than accurate considerations based on a plethora of data [1].

A statement can be true “with some degree of truth” [1], and not just true or false as Boolean logic suggests, the logic on which the modern computer is based on.

Dr. Lutfi Zadeh of the University of California at Berkeley in the 1960s was the first to introduce the concept of fuzzy logic. Fuzzy logic includes 0 and 1 as extreme cases of truth but also incorporates intermediate states of truth [1]. Fuzzy logic resembles the way human brains work.

## 4. Fuzzy neural networks

The development of a fuzzy system with high-performance is not easily accomplished. Several problems arise, including the search of membership functions and appropriate rules, a process which regularly leads in errors. As a result, the learning algorithms were also applied to fuzzy systems. Neural networks, were considered as an alternate way to automate the development of fuzzy systems [6]. The functions of neural networks include but are not limited to process control applications, data analysis and classification, detection of imperfections, and support to decision-making.

**Figure 2.**
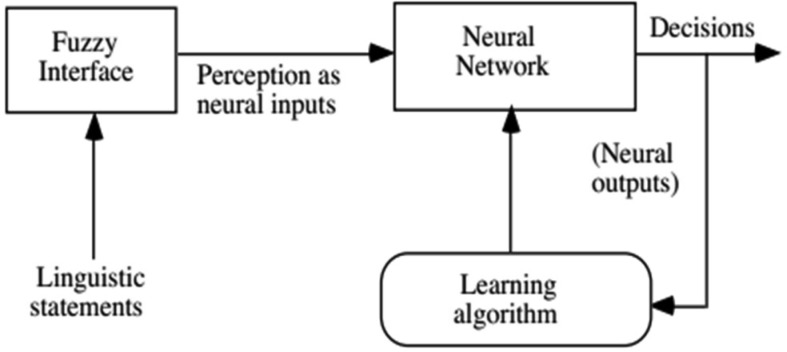
Fuzzy Neuron model.

**Figure 3.**
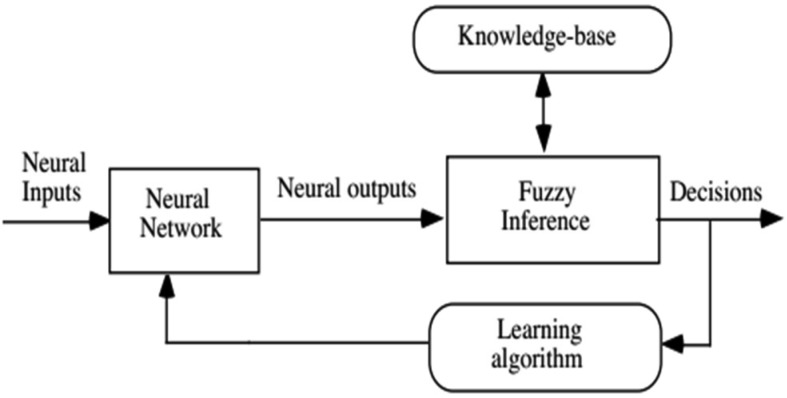
Fuzzy Neuron model.

Neural networks and fuzzy systems can be fused in order to increase their advantages and to decrease their shortcomings. Neural network learning techniques can be utilized in order to substantially reduce the development time of fuzzy systems as well as the cost while improving the performance rates [7]. Figures 2 and 3 present two potential models of fuzzy neural systems. In Figure 2, the fuzzy interface block provides an input vector to a multi-layer neural network as a response to linguistic statements. Subsequently, the neural network is trained to generate required outputs or decisions [8].

In the second case, the fuzzy inference mechanism is determined by a multi-layered neural network.

The computational characteristics of learning offered by neural networks are obtained by fuzzy systems and in return, neural networks receive the interpretation and clarity of systems representation [9]. A fuzzy neural network or neuro-fuzzy system (NFS) utilizes approximation techniques acquired from neural networks, in order to identify parameters of a fuzzy system (i.e., fuzzy sets, fuzzy rules).

## 5. Neuro-Fuzzy systems categories

### 5.1. Cooperative Neuro-Fuzzy system

**Figure 4.**
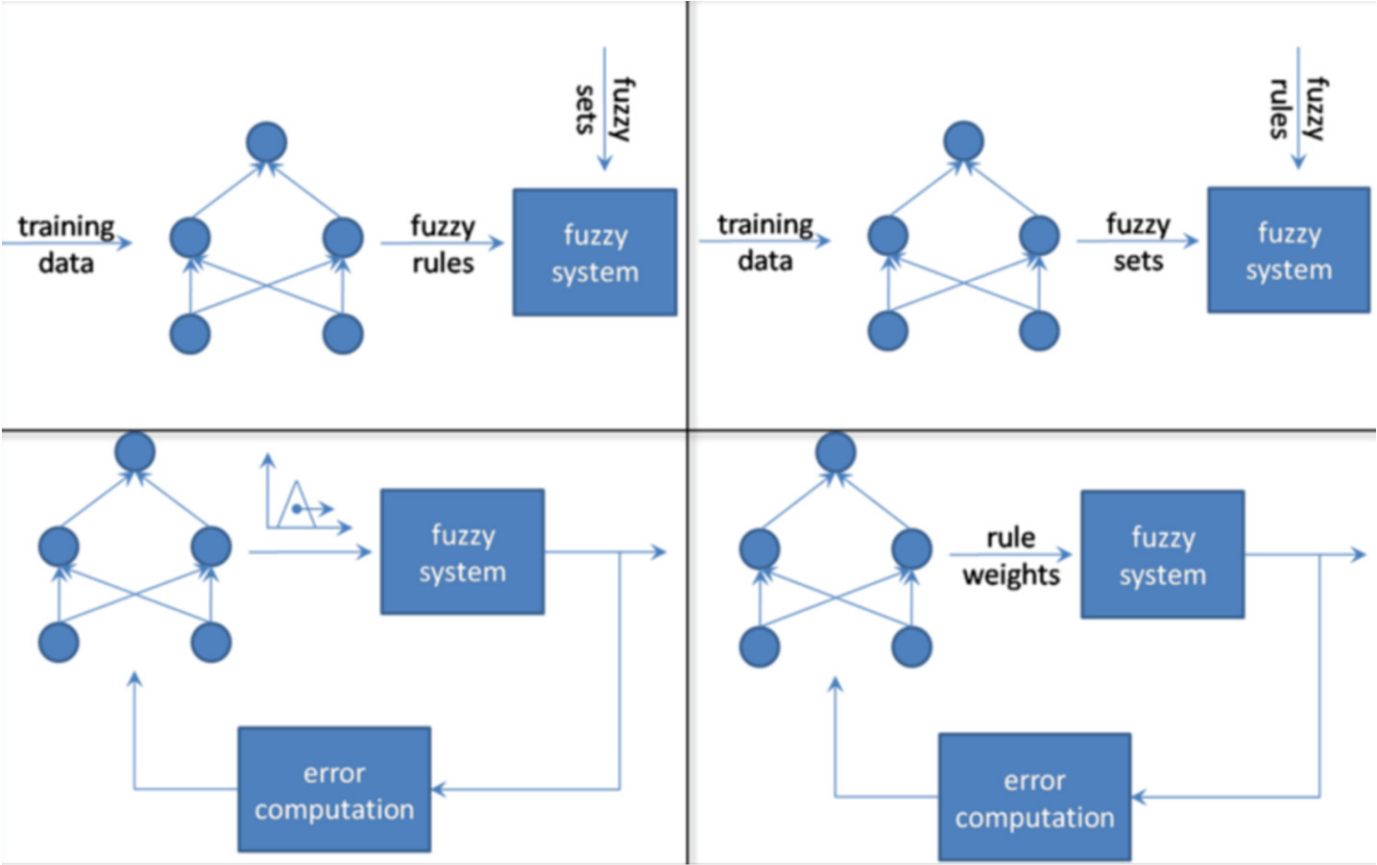
Depicts four different kinds of cooperative fuzzy neural networks.

**Figure 5.**
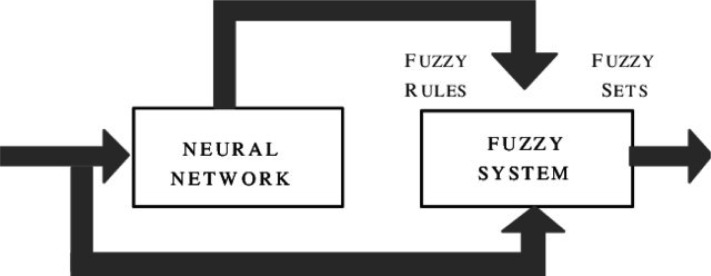
Cooperative Neuro-Fuzzy System (taken from [6]).

For the model of cooperative neural fuzzy systems as shown in Figures 4 and 5, the artificial neural network (ANN) and fuzzy system work independently. The ANN tries to learn the parameters from the fuzzy system, a process that can be performed either offline or online [8].

In the upper left example of Figure 4, the fuzzy rules provided by the training data combined with the fuzzy sets are utilized to form the fuzzy system (offline determination).

In the upper right model of Figure 4, the fuzzy neural network learns the fuzzy sets from the given training data (offline determination). As it is shown in Figure 4, in the lower left neuro-fuzzy case, the fuzzy rules and membership functions must be defined beforehand, in order for the system to learn all membership function parameters online. For the improvement of the learning step, the error has to be measured. In the lower right model, a rule weight which is interpreted as the influence of a rule, is determined for all fuzzy rules by a neural network (both online and offline determination) [8].

A cooperative system only utilizes neural networks in an initial phase. The neural networks using training data, establish sub-blocks of the fuzzy system. Subsequent removal occurs, resulting in the implementation of only the fuzzy system [6].

### 5.2. Concurrent Neuro-Fuzzy system

**Figure 6.**
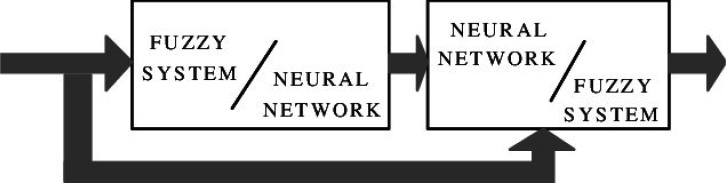
Concurrent Neuro-Fuzzy System (taken from [6]).

In the concurrent neuro-fuzzy system (Figure 6), the neural network and the fuzzy system constantly function in a collective manner, with the neural network pre-processing the inputs of the fuzzy system.

### 5.3. Hybrid Neuro-Fuzzy system

**Figure 7.**
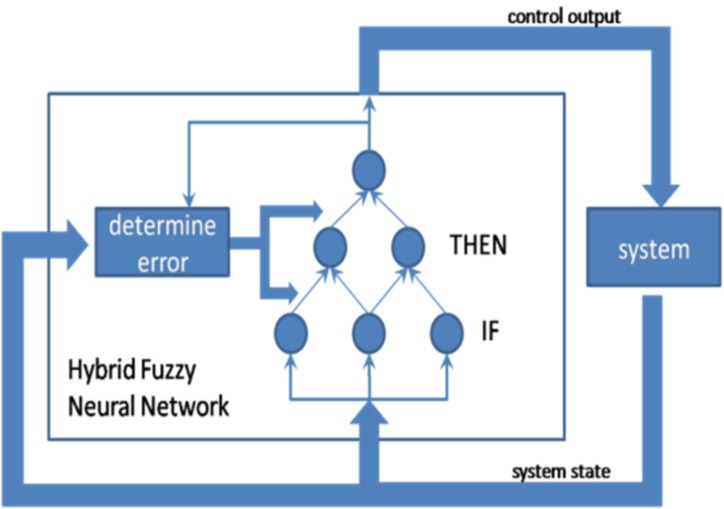
Hybrid FNN.

Hybrid neuro-fuzzy systems (Figure 7) utilize neural networks in order to identify certain parameters of a fuzzy system. In this case, the architecture of hybrid NFS offers a great advantage seeing as the fuzzy system and neural network do not have to communicate with each other. In addition, these systems can learn online and offline [6].

## 6. Fuzzy systems in medicine

Due to their effectiveness, AI (artificial intelligence) techniques, such as fuzzy logic, play a prominent role in the field of medicine. Such methods allow for not only an efficient but also a prompt diagnosis. Guzmán et al., 2019 created a fuzzy classifier in order to perform blood pressure level classification. The main results of this study showed that a type-1 fuzzy inference system or an interval type-2 fuzzy inference system constitute the best architectures to perform said classification [10]. Fuzzy logic has been also applied in order to provide risk assessment for hypertension. Melin, Miramontes and Prado-Arechiga, 2018, designed a model that combined neural networks and fuzzy logic for this purpose. Fuzzy systems were a key part of this study since they regulated the classification uncertainty. This hybrid model provided good results with excellent performance regarding its task [11]. Studies have also shown that fuzzy systems can be applied in Parkinson's diagnosis. Abiyev and Abizade, 2016, proposed a system for Parkinson's disease diagnosis based on the fusion of the fuzzy system and neural networks. The proposed fuzzy neural system (FNS) allows for efficient classification of healthy individuals, a fact that was established through simulation of the system using data obtained from UCI machine learning repository [12].

Another study tested the technique of classifying medical data sets by constructing fuzzy inference systems or fuzzy expert systems. The analysis of data related to Parkinson's yielded a large amount of information. In order to further study and explore the information provided, clinical observations, and disease diagnosis were mathematically translated. Knowledge-based Systems in combination with Data Mining tools and a fuzzy decision maker as well as Artificial Neural Networks Classifiers proved to be useful techniques for mapping clinical data to a numerical data set by exploiting a set of rules [13].

Kaur et al., 2017, described Parkinson's disease using an adaptive neuro-fuzzy technique. According to their results, the adaptive neuro fuzzy expert system showed higher accuracy rates than the fuzzy expert system. In addition, the adaptive neuro fuzzy expert system exhibited higher rate of sensitivity, specificity, and precision when compared to a fuzzy expert system [14].

## 7. Discussion and future work

In this paper, “fuzzy logic” systems which could be used to formalize approximate reasoning in medical diagnostic systems are described. The potential implementation of fuzzy artificial networks in medicine is also analyzed. Authors further work would focus on applying the aforementioned techniques for the establishment of intelligent systems that could be utilized in disease treatment and diagnosis. In more detail, future steps would include the development of a fuzzy expert system that would be utilized in PD diagnosis. This study would include experiments that would evaluate parameters such as accuracy, sensitivity, and specificity.
